# Materia Medica and Therapeutics

**Published:** 1899-11

**Authors:** 


Materia Medica and Therapeutics. An Introduction to the Rational Treatment of Disease. For the use of students and practitioners of medicine. By J. Mitchell Bruce, M. D., F. R. C. P., etc., Physician and Lecturer on Medicine at Charing Cross Hospital, London. New (6th) edition, revised and enlarged. In one 12mo volume of 618 pages. Philadelphia and New York: Lea Brothers & Co. 1899.

The author, in the preface, announces the scope of this work to be chiefly therapeutical, a rational guide to the student and practitioner of medicine in the treatment of disease.

"At the same time the materia medica has not been sacrificed. On the contrary, it will be found to be set forth in detail by the adoption of a natural and concise arrangement, which presents the subject in such a form that it can be quickly appreciated and easily remembered. The author attaches importance to the plan which he has adopted in the description of the special therapeutics, and which consists in systematically tracing the physiological actions and uses of the different drugs in their passage through the body, from their first contact with it locally until they are eliminated in the secretions. In the part of the manual devoted to general therapeutics he has further departed from the ordinary arrangement, by discussing the actions and uses of remedies, not under the headings of artificial groups, but of the physiological systems of the body, digestion, respiration, and the like, so as to conduct the student from facts with which he is familiar to the great principles of treatment."

The book is of convenient size, and this combined with the excellent manner in which the subject is presented, and its moderate price, will commend it to those who wish something on materia medica and therapeutics less formal than a treatise and more complete than a quiz compend. M. J. F.

